# Plasmonic Implanted Nanogrooves for Optical Beaming

**DOI:** 10.1038/s41598-018-37202-5

**Published:** 2019-01-23

**Authors:** Salman Daniel, Prince Bawuah

**Affiliations:** 1 Institute of Photonics, University of Eastern Finland, P.O. Box 111, FI-80101 Joensuu, Finland; 2 Department of Chemical Engineering and Biotechnology, University of Cambridge, Philippa Fawcett Drive, CB3 0AS Cambridge, United Kingdom

## Abstract

Surface plasmon polaritons are electromagnetic surface waves, which, due to their nanoscale nature, are efficiently used for modifying an output of optical field through a metallic nanoslit, e.g., extraordinary optical transmission and beaming of light. Herein, the phenomenon of optical beaming by employing a regular array of semicylinder-shaped grooves around a nanoslit has been investigated based on numerical simulations. By analyzing the behavior of Poynting vectors in near surroundings of the slit, we have successfully demonstrated that grooves which are embedded on the layer at the exit side of the slit produce enhanced directionality of the output light than the unembedded ones. In case of semicylinder-shaped grooves, the calculated intensity of the output beam was 1.5-times, at near and far distances, higher than that of the grating grooves. Our analysis shows that positioning of the groove right at the exit of the slit is crucial for the enhancement of the beaming effect. This is due to the conversion of surface plasmon polaritons into a freely propagating field and the possible excitation of localized surface plasmons because of the presence of nanogroove. Furthermore, the proposed geometries are made of Aluminum, which is a plasmonic material and commonly applied for the fabrication of optical nanostructures. Manipulating of light (beaming, focusing/guiding, and splitting) by nanoslit can be beneficial to several applications such as nano-resolution optical imaging, sensors, and plasmonic circuits.

## Introduction

Surface plasmon polaritons (SPPs) are electromagnetic surface waves that propagate at a metal and a dielectric interface. The SPPs are photon-electron hybrid modes which are tightly confined to the subwavelength surfaces^1^. Surface waves, in general plasmons, are extensively studied in recent years due to their potential applications in, for example, optical sensors^2–4^, waveguides^5–8^, data storage and transformation^9,10^ just to name a few.

Surface plasmons polaritons can be generated by applying nanoridges on the metallic surface, e.g., gratings and slits^11,12^. It has been reported that SPPs can mediate the optical transmission through a nanoslit that is perforated in a metallic sheet^13^. For example, if a slit is surrounded by a regular array of grooves on the input side of the incidence beam of light, the transmission of light is enhanced due to SPPs mediation with the field inside the slit^14,15^. The phenomenon is called an extraordinary optical transmission. With a similar combination of slit as well as an array of structured grooves at the exit side of the slit, the beaming or directionality of the output field can be efficiently enhanced^16^. It is investigated theoretically and experimentally that beaming of light emerges due to the coupling between localized modes of grooves and diffracted wave patterns of the field^16,17^. In recent years, different combinations of slits and 1D grooves are employed for studying the phenomenon of beaming or directionality of light. For example, grating grooves^17–23^, annular aperture array^24^, metasurfaces^25^, dielectric ridges^26^, array of nanoslits^27^ and circular disk^28^ etc.

Although, the role of SPPs is essential for beaming of light, however there is still a need for thorough investigations into this phenomenon, especially the application of various shapes of grooves. This study employs numerical simulations based on finite element method to further probe into the phenomenon of light beaming with a nanoslit surrounded by a regular array of parallel 1D nanostructures of a semicylinder shape. For the sake of comparison, commonly used 1D binary grating grooves are also studied. The employed 1D nanofeatures on the exit side of the slit are divided into two categories: (1) nanostructures are patterned on the surface and (2) nanostructrues are ingrained on the surface. In other words, the 1D undulations on the surface are unembedded in category (1) whereas in category (2), the nanostructures are embedded on the layer. To observed the beaming effects of the two categories of the nanostructures, we analyzed the behavior of Poynting vectors or energy flow in the near surroundings of the nanoslit. It is demonstrated that the beaming of light is strongly enhanced when a nanogroove is placed just at the exit of the slit. It was generally observed that embedded grooves on the layer provide relatively high enhancement in the beam directionality. This study highlights a significant beaming effect of semicylinder-shaped grooves patterned around a nanoslit. It throws more insight into the contribution of both SPPs and localized surface plasmons in the enhancement of light transmission through a slit. The investigation further contributes to the creating of awareness on the significant influence of groove position around the slit on optical beaming. This kind of study of light directionality could be beneficial to several applications such as nano-resolution optical imaging, sensors, and plasmonic circuits.

To further buttress the above observation, the output electric field intensity emerging from the nanoslit was calculated at both near and far distances. The proposed structures are made of aluminum (Al) that is a reliable plasmonic material in the visible spectrum^29^. Unlike the other commonly used plasmonic materials, e.g., Ag and Au^30,31^ etc., Al is less reactive with the environment and commonly applied for fabrication of nanostructures^32,33^.

## Theory and Design

**Figure 1:**
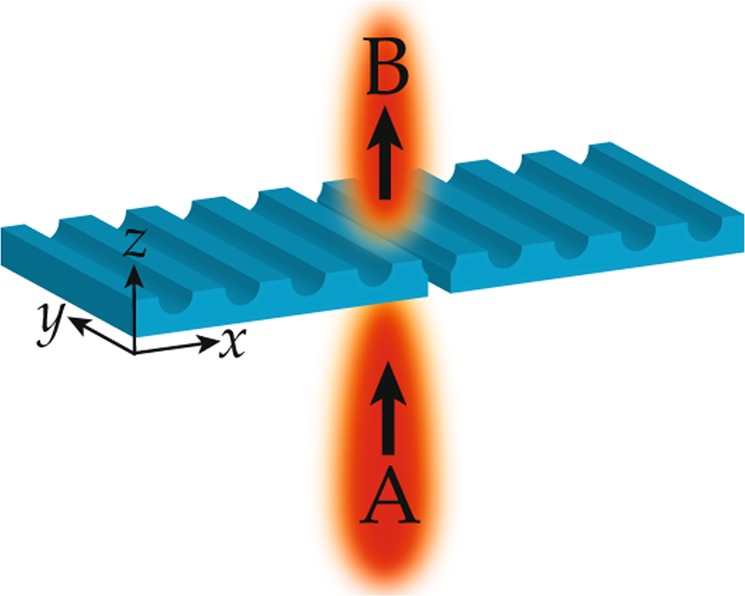
Schematic diagram of beaming of light by a nanoslit that is surrounded by an array of 1D nanostructures. (**A** and **B**) represent incident and transmitted optical fields, respectively.

A schematic approach of the work is shown in Fig. 1. A field **A** that is propagating in z-direction is incident at a nanoslit which is surrounded by a regular array of parallel 1D nanofeatures at the top of the surface. The incidence field of light with a wavelength of 600 nm is polarized along the x-axis (TM); therefore, the component of electric field oscillates in the xz-plane and impinges perpendicularly to the nanoslit. At the slit, the SPPs are excited due to the wavevector phase-matching condition^11^. The SPPs travel through the slit and propagate towards the grooves which are structured next to the slit on the exit side. It is understood that unusual beaming behavior of light through the slit occurs because of the diffraction of these excited SPPs modes at the corrugated metallic surface. The output field emerges like a directional beam, which propagates in the z-direction as represented by **B** in the Fig. 1. Further details about the theory can be found in ref.^16^. The size of the slit (width) is less than half of the wavelength (λ/2) of the incidence light, which dominates the near field effects at the slit^1^. The thickness of the layer which contains a slit is more than the skin depth (~3.7 nm) of the Al film.

**Figure 2:**
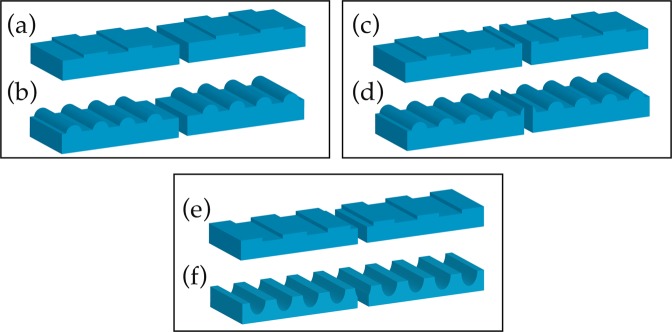
Nanostructure geometries around a nanoslit to investigate the behavior of the output field of light. In (**a,b**), binary grating and semicylinder-shaped nanoridges are structured on top of the surface. (**c,d**) show similar nanofeatures as those in (**a,b**) except the extra half of grating and semicylinder located at the edges of the slit on the exit side. In (**e,f**), the nanostructures are implanted on the top surface of a 300 nm thick layer of Al. The width of the slit is 100 nm. In all cases, a binary grating with width = 200 nm, height = 60 nm and period = 400 nm as well as a semicylinder with radius = 150 nm and period = 400 nm were used.

We employed different slit and 1D nanostructure combinations to study the optical transmission through the nanoslit that is influenced by the SPPs. The geometries are divided into two categories: (1) nanofeatures are patterned at top of the surface around the nanoslit, and (2) grooves are ingrained on the top surface. The configurations are shown in Fig. 2. In Fig. 2(a,b), binary grating and semicylinder-shaped nanoridges are structured on top of the surface. In Fig. 2(c,d), the nanofeatures are similar to those in Fig. 2(a,b) except the presence of a half grating and a semicylinder feature at the edges of the slit on the exit side. In Fig. 2(e,f), the nanostructures are implanted on the top surface of a 300 nm thick layer of Al. In other words, in Fig. 2(e,f) the features are inversed or flipped up-side-down in comparison with Fig. 2(c,d). Notice that the edges of the slit on the exit side in Fig. 2(a,b) are uniform along the x-axis due to the absence of the grooves at the slit site. The size or width of the slit is 100 nm. The optimized parameters of the applied nanostructures, grating and semicylinder features, are given in the caption of the figure.

## Results and Discussion

**Figure 3:**
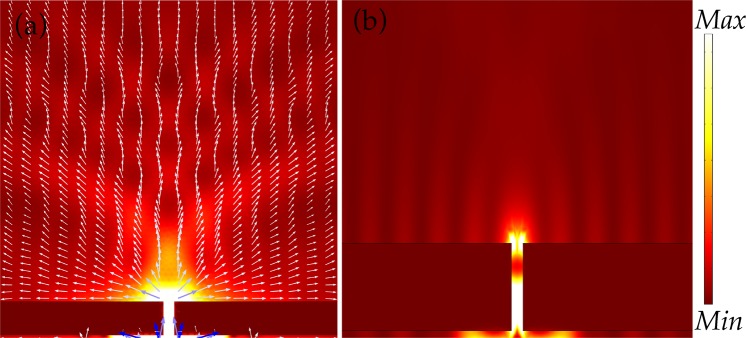
Distribution of energy in near surrounding of a nanoslit. The slit is not surrounded by any nanofeatures. In (**a**), energy flow is plotted wherein white arrows represent the Poynting vectors. In (**b**), electric field intensity |E|^2^ is plotted and ripples on the surface represent the SPPs modes.

In Fig. 3, only a nanoslit without any nanostructures at the top surface is shown. In Fig. 3(a), energy flow is plotted. Clearly, a slit without grooves transmits a relatively low intensity of light in the propagation direction of the incident beam. Rather, energy is accumulated along the top surface in the form of plasmon modes. The energy of the SPPs decays as they travel along the x-axis away from the nanoslit. The white arrows give a close view of distribution of Poynting vectors near the slit. Poynting vector **S** is ***E × B***, wherein ***E*** and ***B*** represent electric and magnetic field, respectively^34^. The length of the arrow represents the magnitude of the energy. The direction of arrows shows that most of the energy of the SPPs flow along the top surface and only a tiny amount of energy (small arrows) is diffracted away from the slit. The arrows in blue color on the incident side of the layer represent the energy of the incidence beam of light. In Fig. 3(b), electric field intensity in the xz-plane (|E|^2^ = E_x_^2^ + E_z_^2^) is plotted to visualize the presence of the SPPs more clearly. The SPPs modes on the metallic layer appear as ripples, and are confined to the surface. Plots of energy that is calculated at the near and the far distance from the slit will be given later.

**Figure 4:**
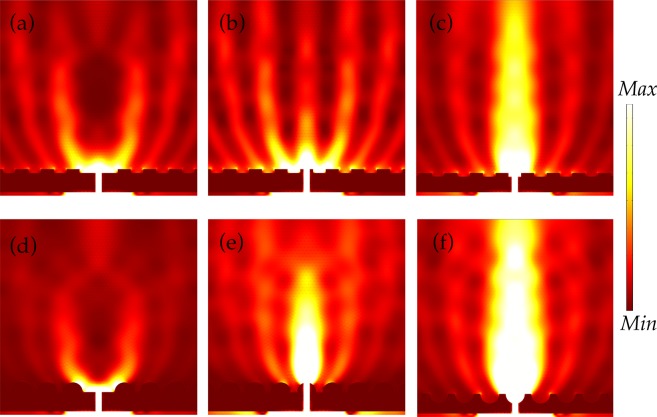
Energy flow or Poynting vectors in near surrounding of a nanoslit on exit side. In (**a** and **b**), grating features are present on top of the exit surface while in (**c**) the features are embedded on the top surface of Al layer. In (**d**, **e** and **f**), semicylinder-shaped grooves are applied on the exit surface with arrangements akin to (**a**, **b** and **c**).

Distribution of energy or Poynting vectors on the exit side of the nanoslit for grating and semicylinder 1D nanostructures that are applied on the surface is shown in Fig. 4. In Fig. 4(a,b), grating features are placed at the top of the exit surface while in Fig. 4(c) grating grooves are embedded on the surface. The field in (a) is mostly confined to near vicinity of the slit. To some extent, the field attains directionality due to the 1D ridges which are placed next to the slit. Nevertheless, directionality is rather weak and beam is split into parts. Diffraction patterns can be observed on the exit side of the slit. In (b), optical beaming is stronger in comparison with (a), which is due to the presence of ridges at the edges of the slit at the exit side. Despite the relatively weak beaming in (b), the sharp ridges gather the energy in the propagation direction. The field is extremely beam-like in (c) where grating grooves are embedded on the surface. Intriguingly, groove that is placed just right at the slit enhanced the beaming effect significantly by aligning the Poynting vectors in the z-direction. This bizarre behavior of the Poynting vectors will be discussed in the later part. Beaming of light emerges because of the coupling between localized modes of grooves and diffracted wave patterns as it is reported by Martín-Moreno^16^.

In Fig. 4(d,e), semicylinder-shaped grooves are placed on top of the surface. The behavior of output field in (d) is approximately the same as in (a). The field in (e) is highly directional and enhanced where the sharp edges of the grooves are present at the exterior edges of the slit. However, it is interesting to notice that embedded grooves in Fig. 4(f) give the strongest optical beaming in the propagating direction. In this case, the semicylinder grooves have high light confining effect. In general, embedded grooves, in (c) and (f), provide relatively high directionality of the output field of light. The output field propagates like a beam in the z-direction. The physical mechanism of beaming of light can be understood by assuming that the SPPs travel through the slit and propagate towards the grooves which are patterned next to the slit on the exit side. The optical beaming through the slit occurs because of the diffraction of these excited SPPs modes at the corrugated metallic surface. The presence of the semicylinder-shaped groove at the exit position of the slit converts the SPPs into freely propagating field^35^, and the shape of the groove aligns the field in a confine manner. The process of SPPs conversion into a propagating field takes place instantaneously with a relatively small decay in the strength of plasmons because of the placement of the nanogroove right at the exit position. It is noticeable that without the presence of nanogroove at the exit of the slit the SPPs travel more and decay substantially before diffraction occurs due to the array of grooves. Furthermore, it has been reported that extraordinary transmission of light through a nanoslit is significantly influenced by excited localized surface plasmons for relatively thick metallic substrates, i.e., the thickness should be in the order of several skin depth of the EM radiation^36^. The skin depth of Al at 600 nm wavelength is c.a. 3.7 nm, hence the designed structure of this study fulfills the above condition, which accounts for the tremendous transmission observed for grooves placed in a close proximity of the slit. In other words, the presence of the groove increases the excitation of localized surface plasmons which could play a role in the enhancement of optical transmission.

**Figure 5:**
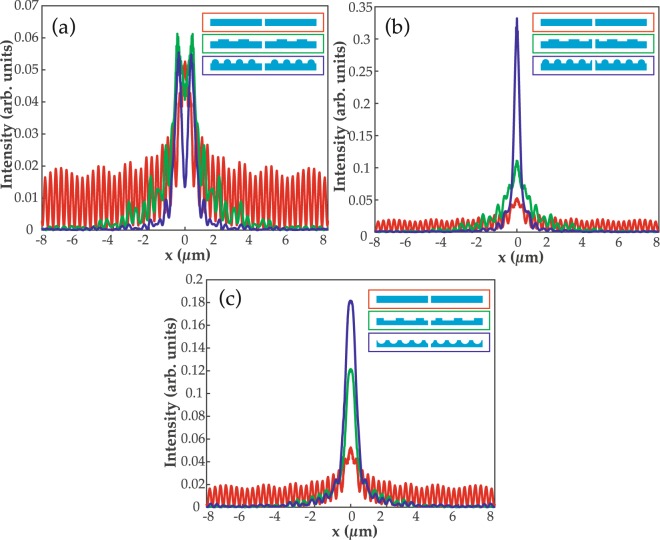
The output electric field intensity through a nanoslit at a near distance of 300 nm. In (**a** and **b**), grating (in green) and semicylinder grooves (in dark blue) are patterned on top of the exit surface while in (**c**) grooves are embedded on the surface. Insets of (**a**, **b** and **c**) represent the applied geometries on the surface. In all three cases, the transmitted intensity through a slit without any grooves on the surface is shown in a red color.

Electric field intensity at a near distance of 300 nm from the exit edges of the slit along the x-axis is calculated. Fig. 5(a,b) shows the results for unembedded grooves that are present on top of the surface. In case (a), the slit is without any grooves (in red color) and most of the energy is confined along the surface in the form of plasmon modes, e.g., like ripples, as shown in Fig. 3(b). The output field through the slit gains a slight directionality due to the grating (green) and semicylinder grooves (dark blue) on the top. However, the maximum portion of the field is diffracted rather than confined in the propagating direction. In Fig. 5(b), the intensity is around 3.5-times higher in the case of semicylinder grooves than grating grooves. The reason behind this enhancement is the presence of sharp edges of the semicylinder groove at the exit of the slit, and it will be discussed later with the help of Poynting vectors in near surroundings of the slit. In Fig. 5(c), the grating and semicylinder grooves are ingrained on the surface and, clearly, the optical beaming intensity is high. The intensity in the case of embedded semicylinder grooves is 1.5-times stronger than that of the embedded grating grooves.

**Figure 6:**
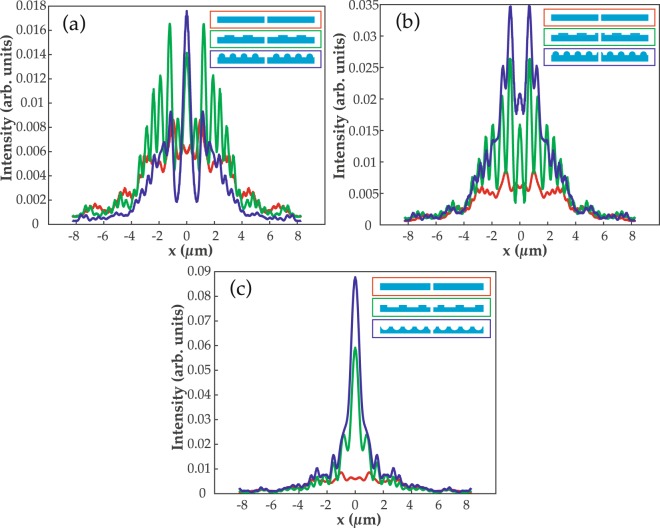
The output electric field intensity through a nanoslit at a far distance of 3 µm. In (**a** and **b**) grating (in green) and semicylinder grooves (in dark blue) are patterned on top of the exit surface while in (**c**) grooves are embedded on the surface. Insets of (**a**, **b** and **c**) represent the applied geometries on the surface. In all three cases, the transmitted intensity through a slit without any grooves on the surface is shown in a red color.

The output electric field intensity through the slit at a far distance of 3 µm is calculated and results are shown in Fig. 6. Akin to Fig. 5, in Fig. 6(a), grating and semicylinder structures are on the top surface. The output field is not behaving like a beam but rather it is diffracted on the exit side of the slit. It is noticeable that diffraction pattern is more visible, which reduces the directionality of the output field of light. In Fig. 6(b), semicylinder grooves display high intensity peaks, however the directionality of the field is relatively low compared to the near field intensity in Fig. 5(b). The intensity with semicylinder grooves is 1.4-times higher than that of the grating grooves. Intriguingly, in Fig. 6(c), the grating and semicylinder-shaped grooves, which are implanted on the exit surface, provide the most confined, directional and high intensity beam at a far distance from the slit. The output intensity due to the semicylinder grooves is 1.5-times higher than the intensity due to the grating grooves. Overall, the intensity in Fig. 6(c) is 5- and 2.5-times higher than in Fig. 6(a,b), respectively. It concludes that the implanted grooves, specially semicylinder ones, are more efficient for producing enhanced beaming of light. In the next section, we will discuss how the positioning and shape of the nanofeatures play a role in optical directionality.

**Figure 7:**
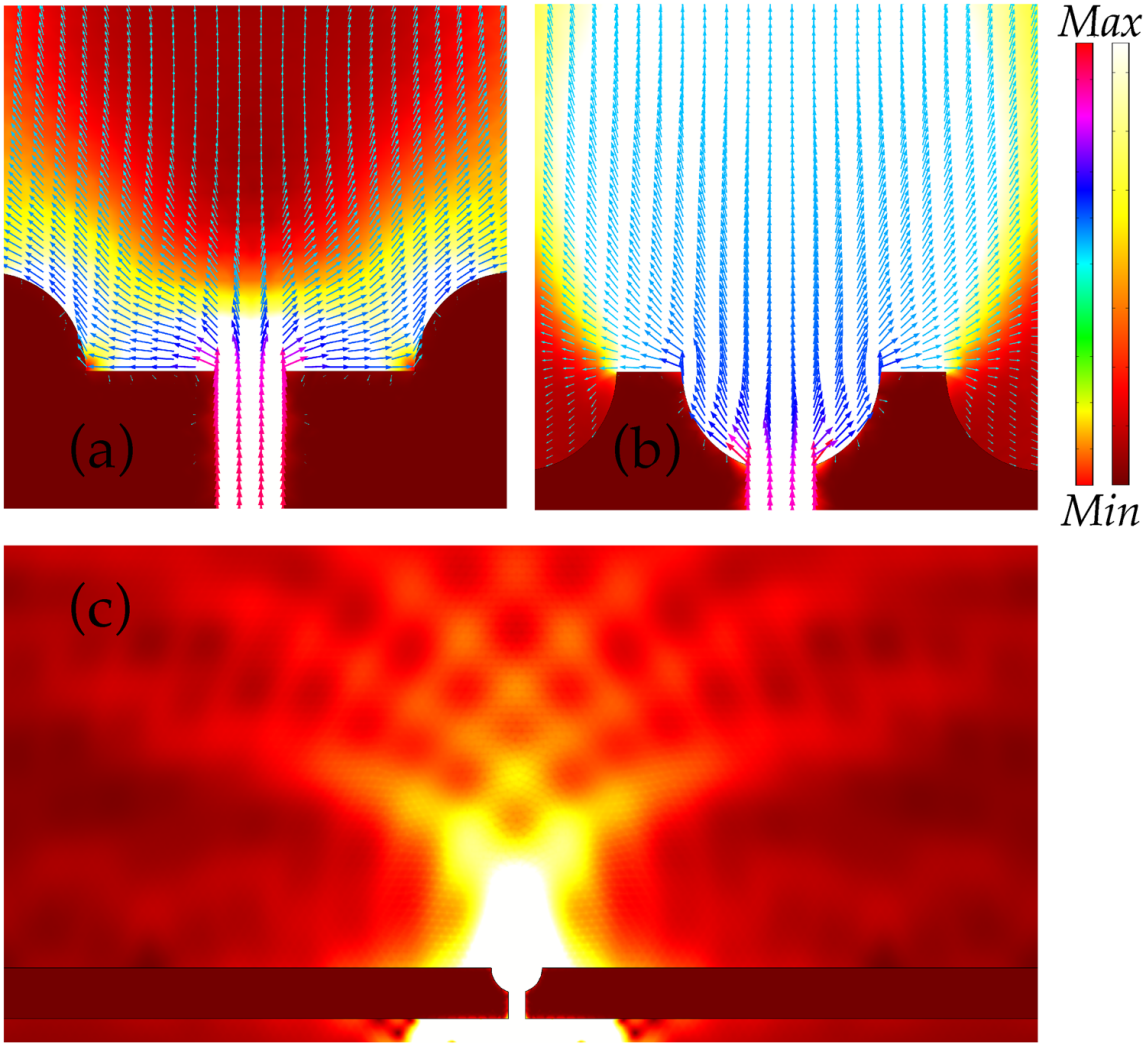
Poynting vectors behavior in near surroundings of a nanoslit. In (**a**), semicylinder-shaped nanofeatures are on the top of the exit surface while in (**b**) features are embedded on the surface. The color bar on the left represents the magnitude of the Poynting vectors while bar on the right shows the electric field intensity. (**c**) shows the behavior of the output field through a nanoslit when only a single nanostructure is planted at the exit of the slit.

The optical field directionality or beaming by a nanoslit in a metallic layer depends on the positioning of grooves that are embedded on the exit surface. In Fig. 7, the behavior of Poynting vectors near the slit is shown. Only a slit with a semicylinder groove is considered in the figure because it gives the maximum beaming of light at a far distance as discussed in the previous section. In Fig. 7(a), semicylinder features are on the surface while in Fig. 7(b) the nanofeatures are embedded or implanted on the surface. Generally, Poynting vectors spread out after passing through the nanoslit but due to the presence of 1D nanostructures, the vectors change their path to provide a directionality as demonstrated in Fig. 7(a). The color coding of the arrows represents the magnitude of the energy. The situation gets more interesting when a groove is planted right at the exit of the slit as shown in Fig. 7(b). Poynting vectors attempt to spread out but after hitting the round boundaries of the nanoridges arrows gain an alignment in the z-direction as they were inside the slit. Poynting vectors that arrive at the exit corners of the semicylinder groove can escape towards horizontal x-axis direction. Nevertheless, this trend of vectors shows that placing of a groove at the exit surface plays important role in the alignment and enhancement of the transmitted beam of light through the slit. Furthermore, it is important to state here that a phenomenon of light directionality occurs with a regular array of parallel grooves which enclose the nanoslit. A single groove at the exit of the slit does not show a beam like behavior considerably. To demonstrate this, in Fig. 7(c) light impinges on the slit which contains a groove (semicylinder) on the exit side. The most of energy is accumulated along the surface rather than propagating in the z-direction.

**Figure 8:**
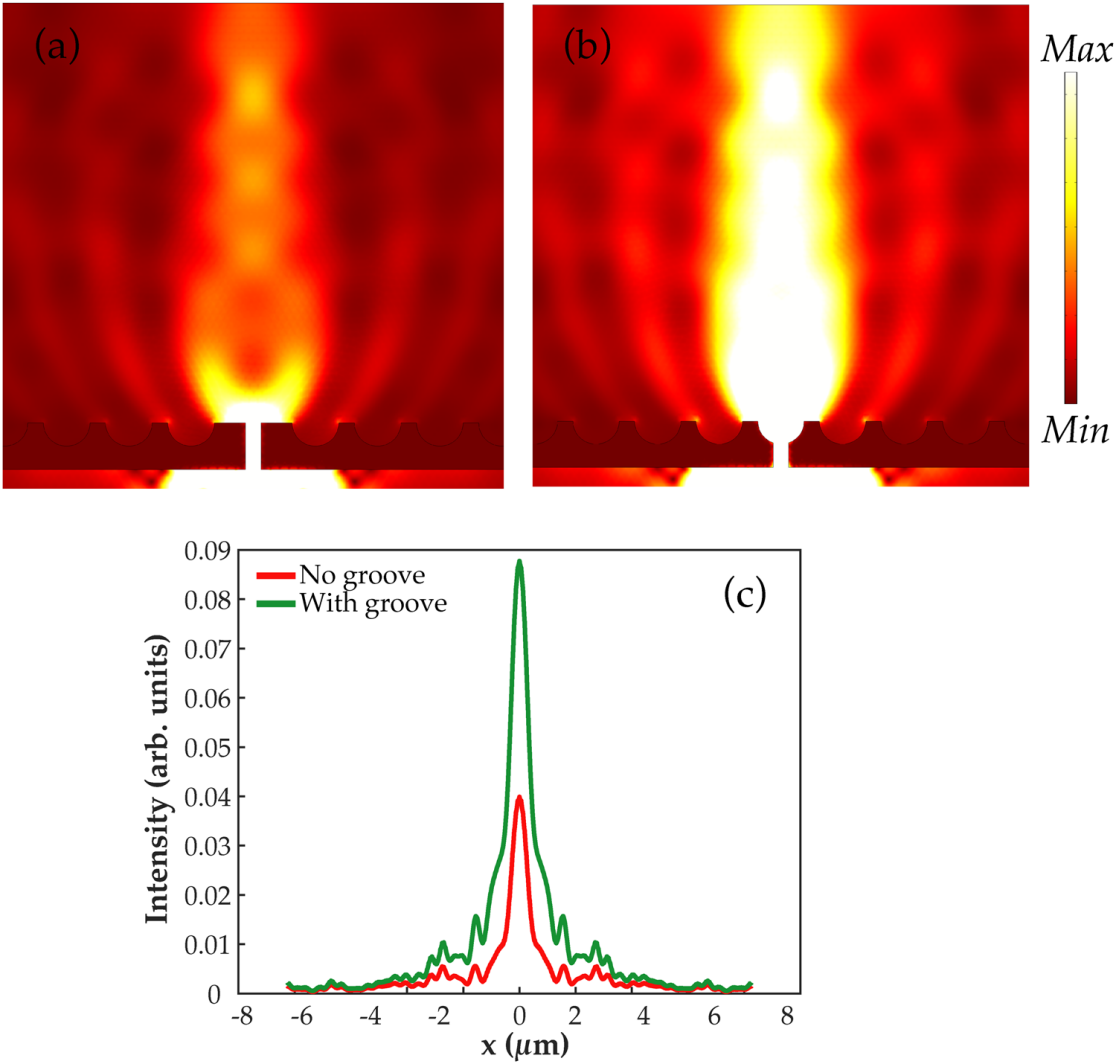
Poynting vectors behavior is presented for semicylinder-shaped grooves that are embedded on the exit surface. In (**a**), slit is without groove at the exit side while in (**b**) a groove is embedded at the exit site of the slit. (**c**) represents an intensity plot for the cases (**a** and **b**).

Figure 8 demonstrates a situation when a nanoslit is (a) without a groove and (b) with a groove at the exit site of the slit. The semicylinder-shaped grooves are embedded on the surface. Clearly, the enhancement of the output beam of light through the slit is higher in Fig. 8(b) than in Fig. 8(a). It explains that a groove presence at the exit site of the slit efficiently increases the intensity of the output beam due to the behavior of the Poynting vectors as discussed previously. Moreover, in Fig. 8(c) intensity is plotted at a far distance of 3 µm from the slit. The intensity is 2.3-times higher for the slit with groove at the exit than that of without the groove.

In general, the proposed 1D grooves which are embedded on the surface at the exit side of the slit show promising response, particularly, semicylinder-shaped features. The beaming of light that emerges due to the mediation of the SPPs well depends on the positioning of the groove at the exit side of the nanoslit. The phenomenon of light beaming appears only because of patterning of surface with an array of parallel grooves on the both sides of the slit. Herein, fabrication of the sample is not a part of this work; however, Al is a commonly used material for manufacturing nanostructrues and therefore we consider that the realization of the proposed nanofeatures is feasible with the available advanced nanolithography techniques^12^.

## Methods

In our model, the simulations of SPP and freely propagating fields on the metallic surface that is perforated with a nanoslit are performed by employing a finite element method (COMSOL Multiphysics). A Gaussian beam with a beam diameter of 1 µm and wavelength of 600 nm propagates along the z-axis, and is incident at the nanoslit that is implanted in Al-layer. The incidence beam of light is polarized along x-axis and it excites SPPs at the silt. The optical material properties of Al are retrieved from Drude-Lorentz model^37^. For example, the relative permittivity value of −47.253 + 16.594i at 600 nm is used. The Al properties are available in COMSOL library. Scattering boundary conditions are applied at the outer boundaries of the model, and the flow of energy or Poynting vectors are calculated in near surroundings of the slit-groove structure.
